# Unveiling the Microstructural Features during Compression of a High-Modulus Mg-15Gd-8Y-6Al-0.3Mn Alloy Reinforced by a Large Volume of Al_2_RE Phases

**DOI:** 10.3390/ma17194784

**Published:** 2024-09-29

**Authors:** Xuhui Feng, Xiaojun Wang, Chao Xu, Hailong Shi, Xuejian Li, Xiaoshi Hu, Zhen Lu, Guohua Fan

**Affiliations:** 1 State Key Laboratory of Advanced Welding and Joining, Harbin Institute of Technology, Harbin 150001, Chinalixuejian@hit.edu.cn (X.L.);; 2 Center for Analysis and Measurement, Harbin Institute of Technology, Harbin 150001, China; 3 Key Laboratory for Light-Weight Materials, Nanjing Tech University, Nanjing 211816, China

**Keywords:** elastic modulus, hot deformation, dynamic recrystallization, texture, microstructure evolution

## Abstract

Magnesium alloys with a high volume fraction of secondary phases exhibit inferior formability. Therefore, investigating their thermal deformation characteristics is critical for optimizing thermal processing techniques. In this work, isothermal compression experiments were performed on a Mg-15Gd-8Y-6Al-0.3Mn alloy with an elastic modulus of 51.3 GPa with a substantial volume of aluminum-rare earth (Al_2_RE) phases. The rheological behavior and microstructural evolution of the material were systematically investigated at varying temperatures (350–500 °C) and strain rates (0.001–1.000 s^−1^). The calculated thermal processing diagram indicates that the unstable region gradually enlarges with increased strain, and all unstable regions appear within the high-strain-rate, low-temperature domain. The ideal thermal processing range of the alloy is 350–500 °C at strain rates ranging from 0.001 to 0.016 s^−1^. Particle-stimulated nucleation and discontinuous dynamic recrystallization are both verified to be responsible for the recrystallized microstructure of the alloy. The recrystallized grains exhibit a relatively random crystallographic orientation. As recrystallization proceeds, the texture gradually transitions from a typical [0001] texture in the compression direction to a random texture accompanied by decreased texture intensity. This work sheds new light on the thermo-mechanical processing of high-modulus Mg alloys, which could help design suitable processing techniques for related materials.

## 1. Introduction

As eco-friendly engineering materials with abundant natural reserves, superior specific strength, and outstanding damping properties, magnesium alloys have been widely used in various industries such as aerospace, transportation, and electronics [1,2,3,4,5]. Numerous efforts to enhance the strength of magnesium alloys have revealed that rare earth–magnesium alloys are the preferred choice of these industries, primarily due to the exceptional mechanical properties and thermal stability of these alloys [6,7,8]. While the mechanical properties of magnesium alloys have been enhanced, the application of magnesium alloys is constrained by their low elastic modulus. The research demonstrates that the modulus can be enhanced by adding a high-modulus secondary phase to magnesium alloys. Conventional methods of introducing external reinforcement phases are limited due to their operational complexity, the emergence of interface issues between the reinforcement phase and the matrix, and problems with solute oxidation [9,10]. In contrast, the in situ synthesis technique introduces a secondary phase that not only strongly bonds with the matrix but also reduces solute oxidation, thereby offering significant advantages [10,11,12,13,14]. Among these, Al_2_Gd and Al_2_Y can be generated in situ, with the secondary Al_2_Y and Al_2_Gd phases exhibiting a modulus of approximately 144 GPa and a melting point of around 1500 °C while maintaining a coherent interface with the α-Mg matrix [15,16]. Consequently, these phases are promising candidates for reinforcement, aiming to produce high-modulus alloys with superior mechanical properties.

Magnesium alloys can be toughened by thermoplastic deformation [17]. Thermal deformation processing effectively refines the grain size and improves the uniformity of the magnesium alloy microstructure [18,19]. Nevertheless, the formability of high volume fraction of second-phase magnesium alloys is generally inferior because the ductility is reduced by the limited independent slip systems of the hexagonal structure of magnesium alloys, and the flow formability is reduced by the externally added hard phase [20,21,22,23]. Moreover, the reinforcement phase added during hot processing increases the material’s resistance to deformation. The highly uneven load distribution between the hardly deformable reinforcement and the matrix causes internal damage such as voids, debonding at stress concentration points, reinforcement fracture, and matrix cracking [24,25]. Investigating the thermal deformation properties is crucial for exploring the mutual interaction between the thermal processing parameters and thermoplastic rheological behavior in magnesium alloys reinforced by a large volume of secondary phases [17,26].

Meanwhile, at elevated temperatures, the deformative characteristics of magnesium alloys often induce dynamic recrystallization (DRX), which is intimately associated with the texture properties of the alloy. DRX aids the grain size refinement and modifies the deformation-induced texture, thus enhancing the mechanical properties of the alloy [27,28]. Given the vital role of DRX in magnesium alloys, the behavior of DRX is worthy of investigation. To reveal the microstructural evolution and DRX behavior of magnesium alloys, the present study investigates the microstructural changes of the alloy during hot forming at varying strain rates and temperatures, laying the groundwork for optimizing the thermal processing parameters of the alloy. Specifically, this study investigates the mechanical properties, the thermal deformation behavior, and the microstructural evolution of the alloy reinforced with a large volume of secondary phases under compression conditions. This work enhances understanding of the flow behavior and recrystallized mechanisms during microstructural evolution in high-modulus magnesium alloys containing a large volume of secondary phase.

## 2. Material and Methods

**Figure 1:**
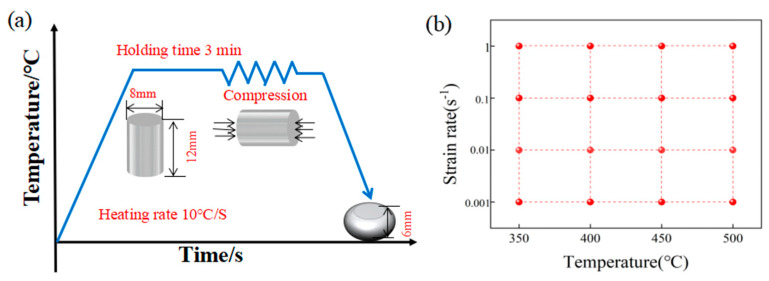
(**a**) Flow of the experimental procedure during compression and (**b**) the experimental parameters.

The alloy was produced by melting Mg-30Y, Mg-30Gd, Mg-10Mn, and Mg-30Al master alloys in an electric furnace under a controlled atmospheric environment with dynamic SF_6_ and a CO_2_ gas flow. The chemical composition (Table 1) was exactly determined using inductively coupled plasma-optical emission spectrometry (ICP-OES). The as-cast alloys were homogenized at 510 °C for 12 h. Cylindrical specimens with a diameter and height of 8 and 12 mm, respectively, were fabricated via wire-cut discharge machining. The test samples were rapidly preheated at approximately 10 °C/s and then maintained at the set temperature for 180 s to ensure uniform heat distribution (Figure 1a). During these processes, the specimens were placed under a vacuum environment to prevent oxidation. As shown in Figure 1b, the experiments were conducted at different temperatures (350 °C, 400 °C, 450 °C, and 500 °C) and strain rates (0.001, 0.010, 0.100, and 1.000 s^−1^). The specimens were compressed to a true strain of 0.7, then rapidly quenched (within 3 s) to retain the microstructures formed during thermal processing. The microstructures were observed under an optical microscope (OM, Leica D4M4M, Wetzlar, Germany), and electron backscatter diffraction (EBSD) measurements were made on a Zeiss SUPRA55 loaded (Jena, Germany) with HKL Channel 5 software. The physical phase was examined using X-ray diffraction (XRD) with a multifunctional X-ray diffractometer over a scanning range of 10–90°. Transmission electron microscopy (TEM) images of the alloys were acquired in a Talos T20 microscope after mechanically thinning the samples to a thickness of 50 μm and punching a 3-mm diameter disk from the thin sheet. The disks were polished to perfection under argon ions using a Gatan precision system. Electron Probe Micro-Analysis (EPMA) was employed to analyze the elemental distribution and chemical composition of the experimental alloys. The fractions of recrystallized grains and secondary phases were measured using Image-Pro Plus 6.0 software. Three relevant images were analyzed for each condition, and the average values were calculated. The elastic modulus of samples sized 4 × 3 × 36 mm^3^ was measured using a Belgian RFDA-HTVP1750 instrument (IMCE LLC, Genk, Belgium). The volume fraction of the second phase was measured using Image-Pro software. The volume fractions were determined by analyzing three different SEM images, and the average value was calculated.

## 3. Results and Discussion

### 3.1. Microstructure of the As-Cast Alloy

**Figure 2:**
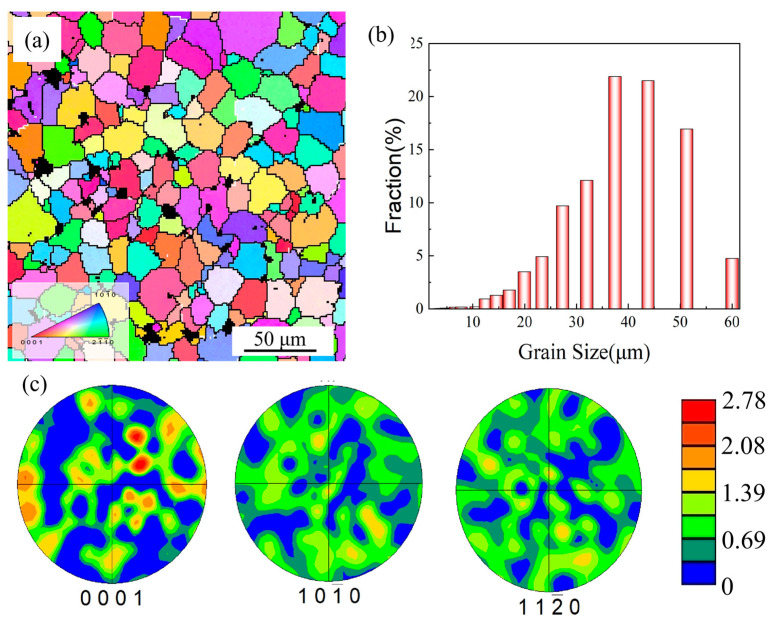
Microstructural characteristics of the as-cast alloy: (**a**) inverse pole figure (IPF) map; (**b**) average grain distribution; (**c**) pole figures of the {0001}, {10-10}, and {11-20} planes.

Figure 2 shows the inverse pole figures (IPFs) of the as-cast alloy. The alloy presents a relatively homogeneous structure with an average grain size of 23.2 μm. The black regions in the IPFs confirm the presence of secondary phases, predominantly along the grain boundaries. The pole figures in the {0001}, {10-10}, and {11-20} planes (Figure 2c) demonstrate a random texture with a maximum intensity value of 2.78.

**Figure 3:**
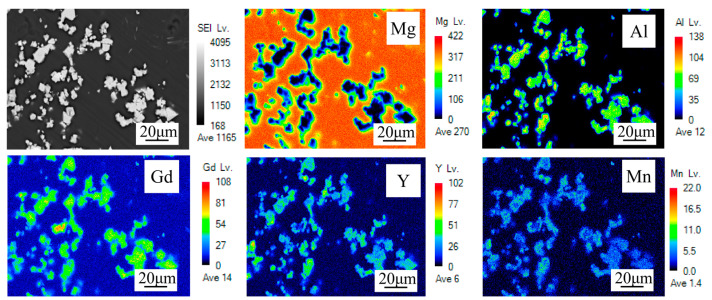
EPMA Results of the as-cast alloy.

**Figure 4:**
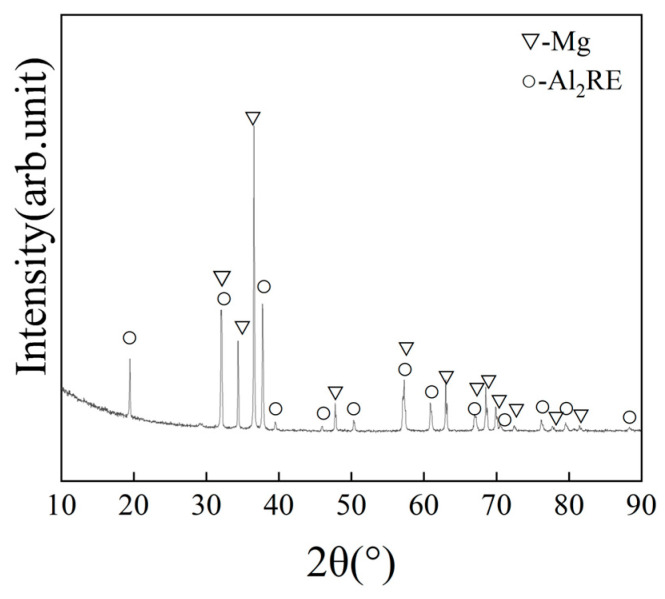
XRD spectrum of the as-cast alloy.

Figure 3 shows the morphology of the secondary phase in the alloy. The secondary phase mainly consists of polygonal particles of varying sizes. Elemental distribution maps reveal that the secondary phase contains Al, Gd, and Y elements. Figure 4 shows the XRD spectrum of the alloy, which only exhibits Al_2_RE (RE = rare earth) and α-Mg diffraction peaks. Therefore, combining the results from Figure 3 and Figure 4, the secondary phase is identified as the Al2RE phase, consistent with previous observations [29,30].

Subsequent measurements of the Al_2_RE phase indicated a volume fraction of 14%. Furthermore, the alloy’s elastic modulus was measured to be 51.3 GPa. Compared to traditional magnesium alloys with an elastic modulus of approximately 45 GPa, this represents an increase of 14%. This finding highlights the critical role of the Al_2_RE phase in enhancing the elastic modulus of magnesium alloys.

### 3.2. True Stress–Strain Curve

**Figure 5:**
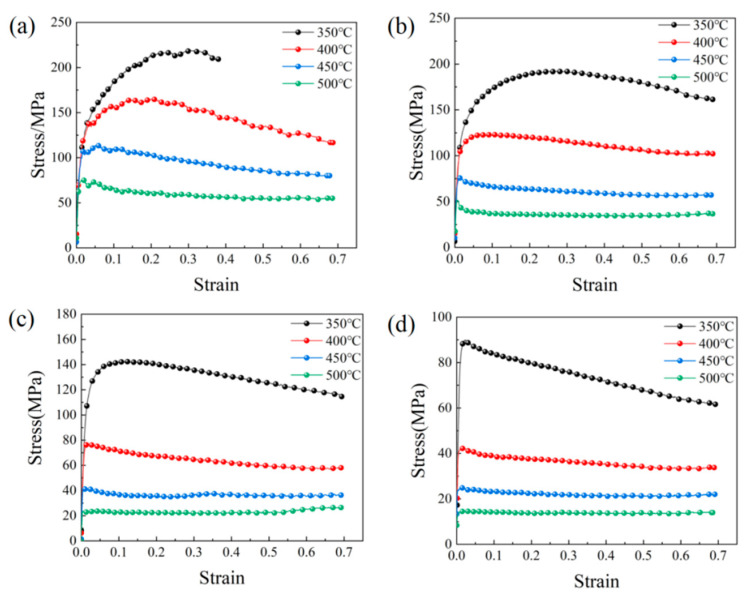
True stress–strain curves at different temperatures and different strain rates: (**a**) 1.000 s^−1^; (**b**) 0.100 s^−1^; (**c**) 0.010 s^−1^; (**d**) 0.001 s^−1^.

The true stress–strain curve of the alloy (Figure 5) can be segmented into three distinct stages. During the initial stage, the stress rises rapidly with strain, and both the number and density of the dislocations rapidly increase. However, the stored energy is insufficient to meet the deformation energy required for DRX and dynamic recovery (DRV). Therefore, this stage is mainly characterized by work hardening. The second stage is characterized by a decrease in and eventual cresting of the strain rate with increasing strain, indicating that work hardening is slower than that in the initial stage. During this stage, the stored energy can satisfy the deformation energy requirements of DRX and DRV. The DRX and DRV processes then begin while some dislocations are annihilated, thereby enhancing the softening. As the strain further increases, the flow stress peaks as the softening rate matches the hardening rate. During the third stage, DRV and DRX continue while the rheological stress in the alloy decreases until it reaches equilibrium. The softening effect produced by the materials plays a major role in this stage [27,31].

The alloy failed prematurely at 350 °C and 1.000 s^−1^, reaching a true strain of approximately 0.4. The noticeable fluctuations in the stress–strain curve at the 1.000 s^−1^ strain rate (Figure 5a) are primarily attributable to the interplay between dynamic softening and strain hardening [32].

**Figure 6:**
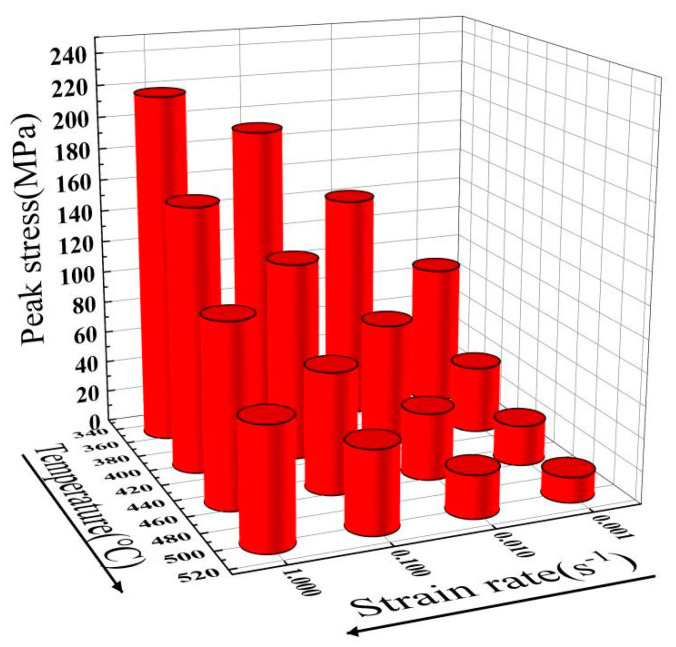
Correlations between peak stress and deformation temperature and strain rate.

Figure 6 shows the correlations between peak stress and the fabricating conditions. At constant temperature, the peak stress diminishes with decreasing strain rate because the durations of DRX and DRV lengthen at lower strain rates, increasing the softening time of the alloy and, thus, declining the peak stress. In addition, when the strain rate is steady, the peak stress is negatively correlated with temperature because higher temperature stimulates the onset of DRX. The activation of various slip systems further enhances the softening effect and decreases the peak stress [33,34,35].

### 3.3. Construction of Constitutive Model

Sellers and McTegart [36] related $\dot{\varepsilon }$ to the parameters of thermoplastic deformation as follows:

(1) $$\dot{\varepsilon }=A[\sin h{\left(\alpha \sigma \right)}^{n}\exp(\frac{Q}{RT})]$$

where *R* denotes the gas constant, *Q* is the activation energy, *T* represents the absolute temperature, *σ* is the flow stress, *α* signifies the stress level, *n* is the stress exponent, and *A* is a constant.

Under low and high stress, Equation (1) is expressed by Equations (2) and (3), respectively:

(2) $$\dot{\varepsilon }=A_{1}\sigma^{n1}\exp(\frac{Q}{RT})$$

(3) $$\dot{\varepsilon }=A_{2}\exp(\beta \sigma )\exp(\frac{Q}{RT})$$

where *A*_1_, *A*_2_, *n*_1_, and *β* are material constants. From Equations (2) and (3), we can infer that *α* equals *β* divided by *n*_1_.

Taking the natural logarithms of both sides of Equations (1)–(3), we obtain Equations (4)–(6) as follows:

(4) $$\ln\dot{\varepsilon }=\ln A_{1}+n_{1}\ln\sigma -\frac{Q}{RT}$$

(5) $$\ln\dot{\varepsilon }=\ln A_{2}+\beta \sigma -\frac{Q}{RT}$$

(6) $$\ln\dot{\varepsilon }=n\ln[\sinh(\alpha \sigma )]+\ln A-\frac{Q}{RT}$$

**Figure 7:**
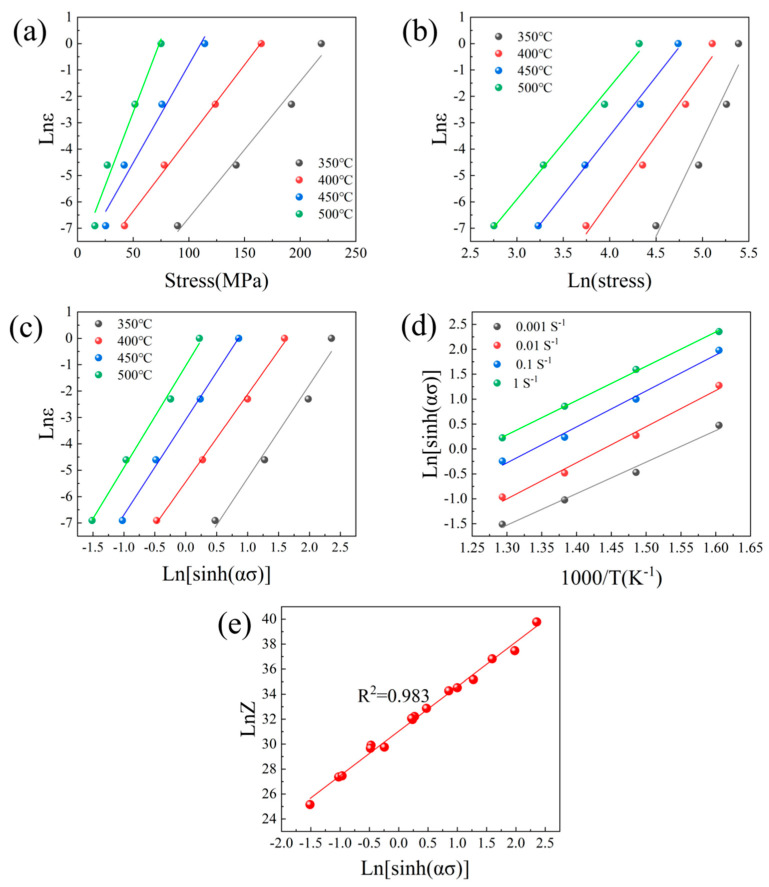
Pairwise parameter relations: (**a**) ln*σp*-ln*ε*; (**b**) In*σp*-ln*ε*; (**c**) ln*ε*-ln[sinh(*ασ*)]; (**d**) 1/*T*-ln[sinh(*ασ*)]; (**e**) ln[sinh(*ασ*)]-ln*Z.*

Fitting the ln*ε*-ln*σ* and ln*ε-σ* relationships (Equations (4) and (5), respectively), the slopes *n*_1_ and *β* were determined as 5.2451 and 0.0791, respectively (Figure 7a,b), and *α* was estimated as *β*/*n*_1_ = 0.0139. Furthermore, fitting the *ε*-ln[sinh(*ασ*)] relationship, *n* was determined as 3.5840 (Figure 7c).

*Q* can be expressed as a function of 1/*T* and ln[sinh(*ασ*)] (Equation (7)). The slope of Figure 7d gives *Q*/*Rn*. Substituting the obtained data for *n* and *R*, *Q* was obtained as 206.1 KJ/mol:

(7) $$Q=Rn\frac{d\left\{\ln\left[\sinh(\alpha \sigma )\right]\right\}}{d(1/T)}$$

The Q value of the alloy is higher than that of pure magnesium (135 kJ/mol) [37] and several Mg-RE alloys, including Mg-5Al-0.6Sc (144.58 kJ/mol) [38] and Mg-2Zn-1Al-0.2RE (144.58 kJ/mol) [39]. This increase is primarily attributed to the substantial presence of Gd and Y atoms, which effectively generate a solute drag effect. These elements hinder dislocation movement by promoting solute atom clustering. Furthermore, the Al_2_RE phase formed within the alloy also contributes to obstructing dislocation motion, resulting in a higher Q value than the aforementioned alloys.

Using the Zener–Hollomon parameter *Z* [40], the flow stress is found to be influenced by the strain rate and deformation temperature:

(8) $$Z=\dot{\varepsilon }\exp(\frac{Q}{RT})=A{\left[\sinh(\alpha \sigma )\right]}^{n}$$

Taking the natural logarithm of both sides of Equation (8), we get

(9) $$\ln Z=\ln\dot{\varepsilon }+\frac{Q}{RT}=\ln A+n\ln[\sinh(\alpha \sigma )]$$

Substituting various deformation temperatures *T*, different strain rates, and the calculated activation energy *Q* into Equation (8), *Z* can be obtained under different compression conditions. Subsequently, the slope of the ln[sinh(*ασ*)] versus ln*Z* plot can be obtained. From the *y*-intercept of this plot (ln*A* in Figure 7e), *A* was determined as 3.0052 × 10^13^. The structural factors of the alloy were then calculated, and the results are presented in Table 2. 

Finally, the constitutive equation is obtained as follows:

(10) $$\dot{\varepsilon }=3.0052\times 10^{13}{\left[\sinh(0.0139\sigma )\right]}^{3.5840}\times \exp(-\frac{206.1}{RT})$$

### 3.4. Construction of Thermal Processing Diagrams

To clarify the processing range of the material, a thermal processing diagram was established using the dynamic material model [41]. In this calculation, the thermally deformed material was considered as a nonlinear energy-loss unit and the energy *P* input to the system was divided into two bodies: dissipation of the plastic deformation *G* and dissipation of the control *J* [41,42,43]:

(11) $$P=\sigma \dot{\varepsilon }=G+J=\int_{O}^{\dot{\varepsilon }}\sigma d\dot{\varepsilon }+\int_{O}^{\dot{\varepsilon }}\dot{\varepsilon }d\sigma$$

The ratio of these two energies during deformation is expressed as the strain rate *m*:

(12) $$m=\frac{\partial J}{\partial G}=\frac{\partial \ln\sigma }{\partial \ln\dot{\varepsilon }}$$

The power dissipation factor *η*, which specifies the ratio of the energy *J* dissipated by organizational changes during deformation to the linearly dissipated energy *J*_max_, is calculated as

(13) $$\eta =\frac{1}{J_{\max}}=\frac{2m}{m+1}$$

The parameter *η* signifies the intrinsic response of the different microscopic mechanisms acting on the machined part within a given temperature and strain range.

According to Ziegler’s maximum value principle, rheological instability occurs when

(14) $$\xi \left(\dot{\varepsilon }\right)=\frac{\partial \ln\left(\frac{m}{m+1}\right)}{\left(\ln\dot{\varepsilon }\right)}+m<0$$

**Figure 8:**
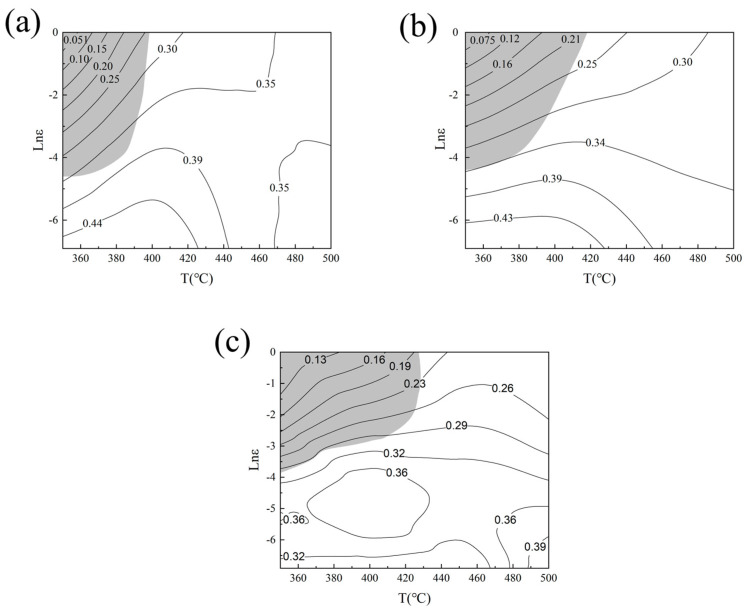
Thermal processing diagrams of the alloy at various degrees of deformation: (**a**) 0.3; (**b**) 0.5; (**c**) 0.7. The magnitudes of the contour lines denote the power dissipation factors.

Figure 8 plots the thermal processing diagrams determined through the above method at true strains of 0.3, 0.5, and 0.7. A higher dissipation factor (higher-magnitude contour lines in the figure) implies a greater proportion of energy used in material transformation. Magnesium alloys are suitable for hot working, and areas with high power loss factors should be selected as much as possible when determining the processing technology [44]. The instability region (gray region in Figure 8) should be avoided during processing. 

As shown in Figure 8, the instability interval of the alloy tends to increase with increasing over the strain range 0.3–0.7. More specifically, the instability interval is roughly [350–398 °C, 0.010–1.000 s^−1^] at a strain of 0.3 and [350–419 °C, 0.013–1.000 s^−1^] at a strain of 0.5. When the strain variable is set to 0.7, the instability interval is approximately [350–429 °C, 0.020–1.000 s^−1^]. The instability region is characterized by a lower power dissipation factor *η* than in other regions. During deformation, most of the energy assimilated by the material is converted to work and heat, leaving insufficient energy for structural evolution. The end result is machining instability. By superimposing and comparing the thermal processing maps at three different strains and referring to previous studies, it was shown that when η > 0.3, it represents the optimal processing zone for the material [38]. Drawing from these findings, the preferred processing window for the alloy is [350–500 °C and 0.001–0.016 s^−1^].

**Figure 9:**
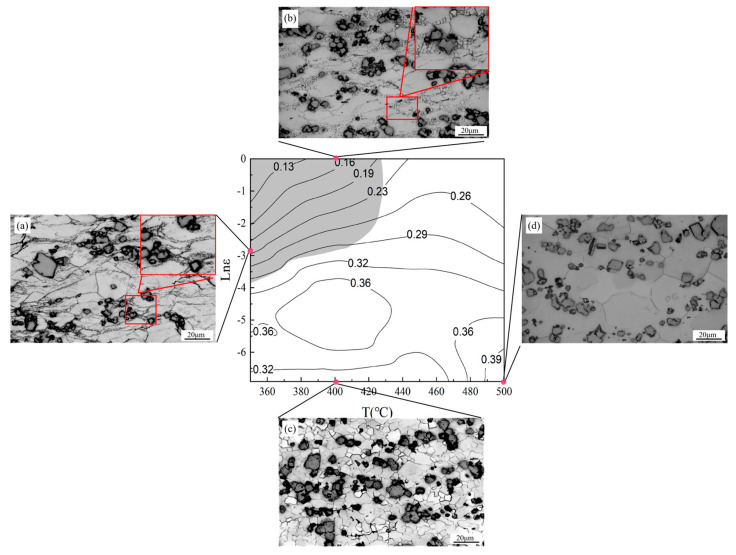
Thermal processing diagram of the alloy at a strain of 0.7 and optical micrographs: (**a**) 350 °C, 0.100 s^−1^; (**b**) 400 °C, 1.000 s^−1^; (**c**) 400 °C, 0.001 s^−1^; (**d**) 500 °C, 0.001 s^−1^.

Four locations on the thermal processing diagram at a strain of 0.7 (a: 350 °C, 0.100 s^−1^; b: 400 °C, 1.000 s^−1^; c: 400 °C, 0.001 s^−1^; d: 500 °C, 0.001 s^−1^) were selected for further investigation. The evaluation focused on the correlation between the thermal processing diagram and the resulting microstructural characteristics (Figure 9). When deformed at 350 °C, 0.100 s^−1^ and at 400 °C, 1.000 s^−1^, the structure was organized into prominent streamlined deformation bands. The primitive grains were compressed and flattened, accompanied by an obvious outward bowing of the grain boundaries. It can be seen that shorter deformation time and lower temperature delay the occurrence of DRX and the release of stress concentration at the grain boundaries [45,46,47]. Consequently, local rheology and destabilization occur in zones A and B in the thermal processing diagrams. Figure 9c,d shows the microstructures in the optimum processing region at 400 °C, 0.001 s^−1^ and at 500 °C, 0.001 s^−1^. The original deformation grain boundaries have begun to disappear and the initially deformed grains have been replaced by DRXed grains.

### 3.5. Microstructure Evolution

**Figure 10:**
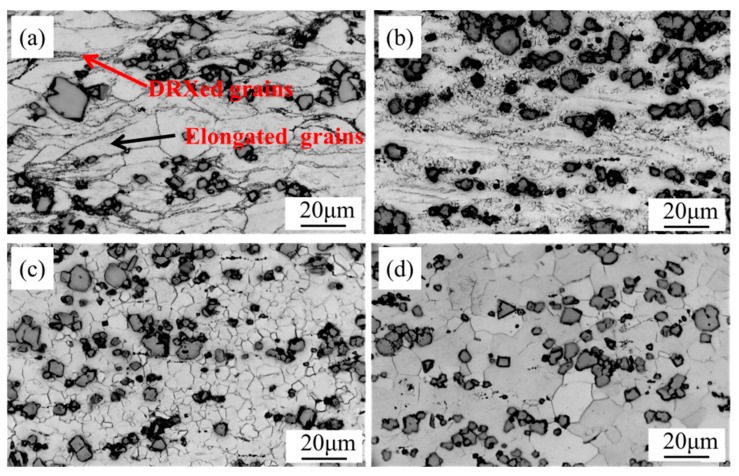
Optical micrographs of the alloy at 0.100 s^−1^ and different temperatures: (**a**) 350 °C; (**b**) 400 °C; (**c**) 450 °C; (**d**) 500 °C.

**Figure 11:**
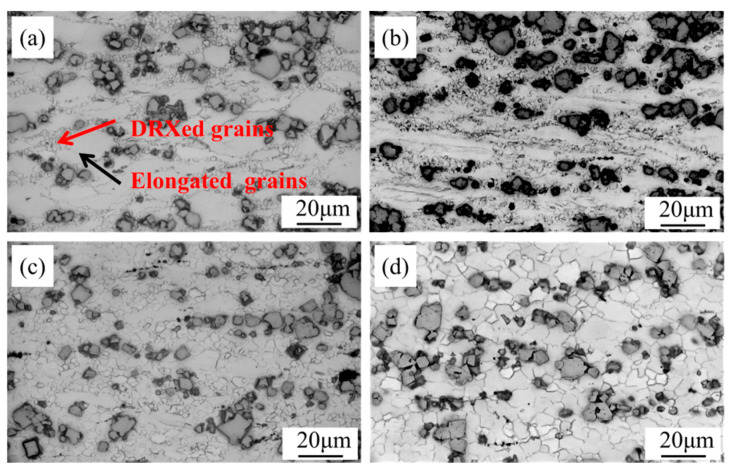
Optical micrographs of the alloy at 400 °C and different strain rates: (**a**) 1.000 s^−1^; (**b**) 0.100 s^−1^; (**c**) 0.010 s^−1^; (**d**) 0.001 s^−1^.

The microstructural evolutions in the alloy were observed at different deformation temperatures under the same uniform strain rate (0.100 s^−1^) (Figure 10) and under different strain rates at the same steady-state temperature (400 °C) (Figure 11). Both temperature and strain rate largely influenced the DRX. Higher processing temperature correlates with a marked increase in the extent of DRX, itself characterized by increased average grain size of the recrystallized microstructure. Upon detailed examination, as the temperature increased from 350–400 °C to 450–500 °C, the DRX ratio increased from 9.3% to 43.1% to 98.2% to 100%. Thermal elevation notably augmented the grain size of the DRXed microstructure. At 350 °C, 400 °C, 450 °C, and 500 °C, the measured dimensions of the recrystallized (DRXed) grains were 0.4, 1.4, 4.1, and 10.8 μm, respectively. Reducing the deformation rate from 1.000 to 0.001 s^−1^ similarly increases the volume fraction of DRX and enlarges the DRXed grains. At strains of 1.000, 0.100, 0.010, and 0.001 s^−1^, the DRX ratios were 15.3%, 43.1%, 94.5%, and 100%, respectively, with corresponding DRXed grain sizes of 1.2, 1.4, 2.1, and 4.6 μm, respectively. At higher deformation temperatures and lower strain rates, the mean grain size and DRX ratio were enhanced through the provision of sufficient time and energy for the free growth of DRXed grains [46,47].

**Figure 12:**
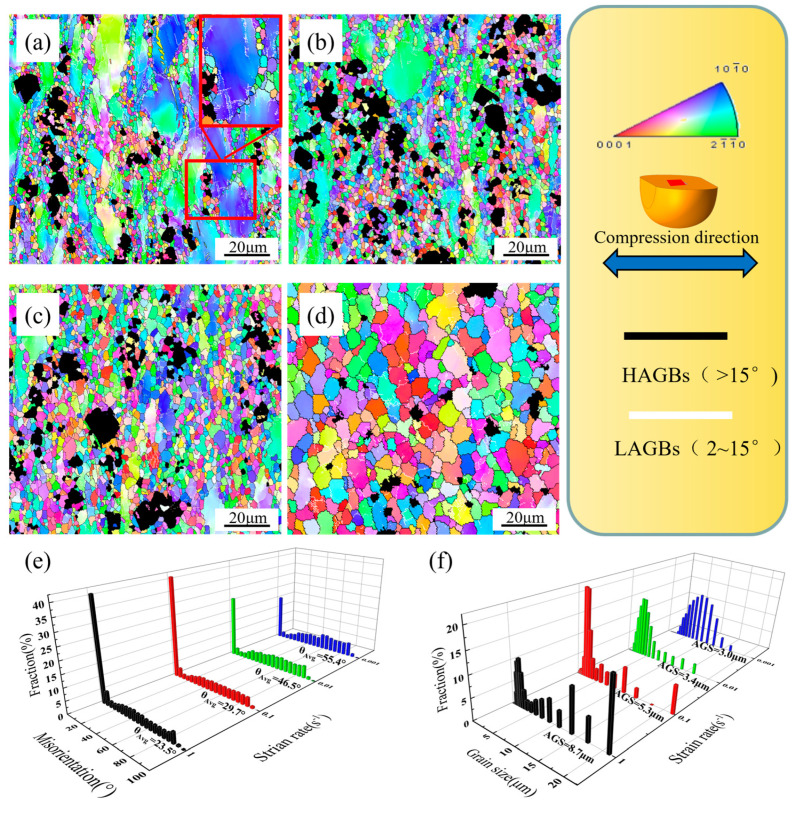
Inverse pole figure (IPF) maps of the alloy under various strain rates at 400 °C: (**a**) 1.000 s^−1^; (**b**) 0.100 s^−1^; (**c**) 0.010 s^−1^; (**d**) 0.001 s^−1^; (**e**) misorientation angle distributions; and (**f**) grain size distributions. The white and black lines indicate the low-angle (2–15°) and high-angle (>15°) grain boundaries, respectively.

To determine the effect of recrystallization on the alloy’s microstructure and texture, samples compressed to a true strain of 0.7 at 400 °C under different strain rates were examined using EBSD. Figure 12 displays the IPF maps, distributions of misorientation angles, and grain size distribution under various strain rates at 400 °C. The evolution of the misorientation angles strongly correlates with the degree of DRX. A larger mean misorientation angle is positively correlated with DRX degree. At strain rates of 1.000, 0.100, 0.010, and 0.001 s^−1^, the average misorientation angles were 23.5°, 29.7°, 46.5°, and 55.4°, respectively, indicating that the DRX fraction increases with decreasing strain rate and is maximized at 0.001 s^−1^, consistent with the previous conclusion. Reducing the strain rate also decreased the grain size, from 8.7 μm at 1.000 s^−1^ to 3.0 μm at 0.001 s^−1^.

As shown in Figure 12, DRX occurs either along the serrated grain boundaries or at the intersections of the initial grains. Grain boundary protrusion is a fundamental characteristic of the discontinuous DRX (DDRX) mechanism, primarily driven by differences in grain boundary dislocation densities resulting from nonuniform strains generated during deformation. Dislocations at grain boundaries with varying densities are prone to slippage, leading to variations in the migration distances of the original grain boundaries and local protrusions of the grain boundaries toward neighboring grains. Facilitated by the activation of additional slip systems, low-angle grain boundaries (LAGBs) form between the parent grain and the protruding part. The resulting substructure comprises the original protruding HAGB and the newly formed LAGBs. Through subsequent rotary motion and grain boundary migration, this substance transforms into DRXed grains [31].

Near the secondary phase, the ratio of DRXed grains considerably overtakes that of the surrounding matrix, demonstrating that the secondary Al_2_RE phase promotes DRX nucleation. As is widely known, nondeformable particles can restrain the flow of the surrounding substance during deformation [48]. Stress accumulation in the vicinity of these particles induces high-density dislocations; the consequent changes in orientation create specific regions called particle deformation zones (PDZs) within the material [31]. During this experiment, the Al_2_RE phase with a higher elastic modulus than the matrix resisted the flow of the matrix during deformation and allowed the formation of PDZs around the Al_2_RE phase. Previous studies have indicated that when the dimension of the secondary phase is less than 1 μm, it tends to hinder recrystallization, whereas diameters greater than 1 μm can boost DRX nucleation [48]. In our investigation, the Al_2_RE particles were considered as potential DRX sites because their diameters surpassed the critical size for stimulating recrystallization nucleation.

**Figure 13:**
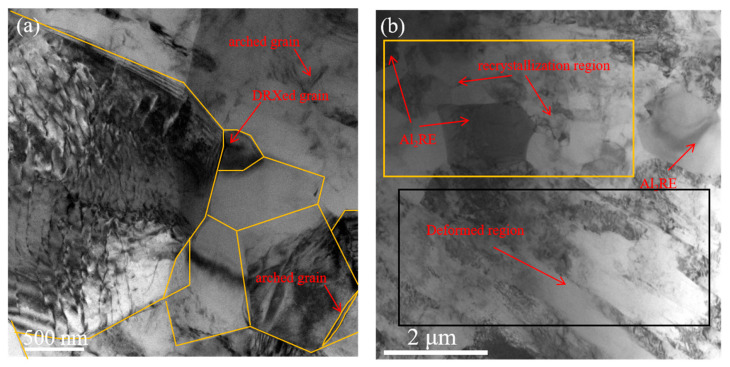
TEM images of the alloy at 400 °C and a strain rate of 1 s^−1^: (**a**) typical microstructure of discontinuous dynamic recrystallization; (**b**) typical microstructure of particle-stimulated nucleation.

The TEM images in Figure 13 clarify the formation of newly DRXed grains at triple junctions and the bending of the grain boundaries. This observation is consistent with the nucleation characteristics of DDRX. Partially arched grain boundaries are also clarified in Figure 13a. Small deformations are nonuniform and lead to notable differences in dislocation density between neighboring grains. Consequently, a small section of the grain boundary bends abruptly toward the side with higher dislocation density, forming an arched segment. The growing arched segment eventually becomes non-hemispherical, and the crystal core grows automatically. This nucleation characteristic is also associated with DDRX, which alleviates the stress concentration resulting from grain boundary sliding and promotes the coordination of grain boundary sliding. Numerous DRXed grains appear near the Al_2_RE phase, and deformed microstructures were observed at locations distanced from the Al_2_RE phase (Figure 13b), further confirming that the Al_2_RE phase promotes DRX. Hence, particle-stimulated nucleation and discontinuous dynamic recrystallization are both verified to be responsible for the DRXed microstructure of the alloy, aligning with the results in Figure 12.

**Figure 14:**
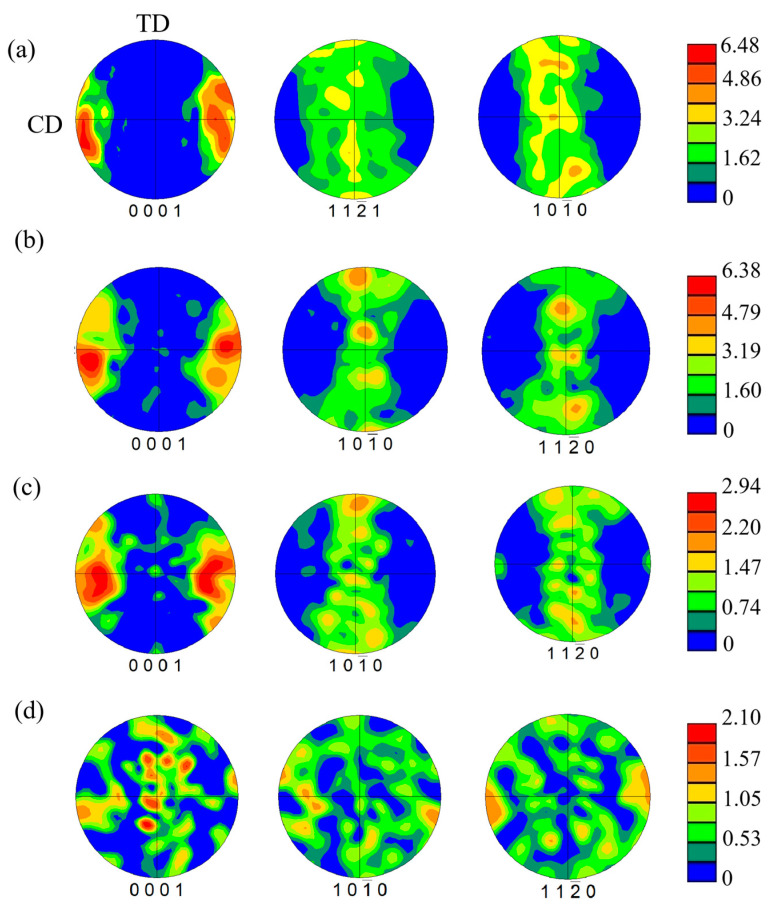
Pole figures of the alloy at 400 °C under different strain rates: (**a**) 1.000 s^−1^; (**b**) 0.100 s^−1^; (**c**) 0.010 s^−1^; (**d**) 0.001 s^−1^.

Figure 14 shows the pole figures of the alloy under different compression strain rates. Within the 0.010–1.000 s^−1^ range of strain rates, the alloys exhibit a typical compression texture with an orientation of <0001>//CD, where CD denotes the compression direction. The texture becomes more disordered at lower strain rates, and the pole density points are randomly distributed at a strain rate of 0.001 s^−1^. Decreasing the strain rate also decreased the overall intensity of the texture: the maximum texture intensity diminished from 6.48 at 1.000 s^−1^ to 2.10 at 0.001 s^−1^.

**Figure 15:**
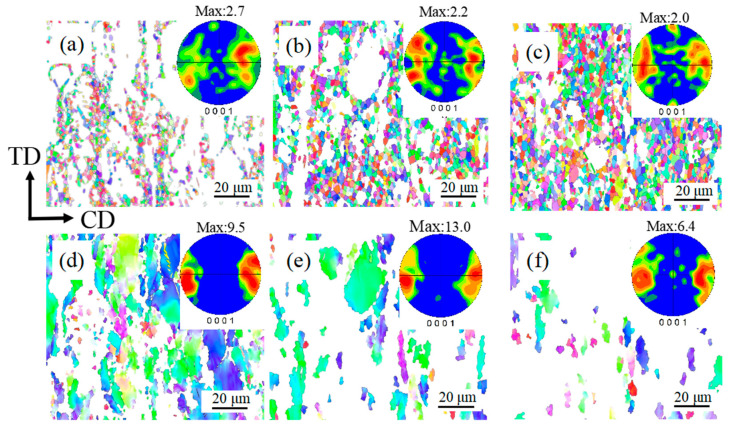
IPF maps and (0001) poles of the alloy after deformation at 450 °C: dynamically DRXed grains at (**a**) 1.000 s^−1^; (**b**) 0.100 s^−1^; (**c**) 0.010 s^−1^; deformed grains at (**d**) 1.000 s^−1^; (**e**) 0.100 s^−1^; (**f**) 0.010 s^−1^. CD and TD represent the compression and transverse directions, respectively.

The degree of texture variation is strongly related to DRX occurrence, as the presence of new grains alters the entire orientation. The impact of DRXed grains on the recrystallization process was analyzed on a partially recrystallized alloy sample. Many previous studies have considered that the grain orientation spread (GOS) of DRXed grains in magnesium alloys is below 2.0. However, a GOS threshold of 2.0 is useful only for assessments of “recently recrystallized” grains [39]. Some equiaxed grains were also identified as deformed grains, suggesting their deformation after early recrystallization during the deformation process. Although these grains are classified as deformed grains in Figure 15, their strain levels are relatively low [39].

As shown in Figure 15, the deformed grains in an incompletely DRXed alloy tend to align along the <0001> direction, and their texture strength exceeds the overall texture strength. Under a strain rate of 1.000 s^−1^, the ratio of DRX is low, and the DRXed grains make small contributions to the overall crystal orientation. The pole distribution of the DRXed grains differs from that of the overall texture, which is dominated by the texture of the deformed grains. At strain rates of 0.100 and 0.010 s^−1^, the ratio of DRX increases, and the DRXed grain structure more substantially contributes to the overall composition. In this case, the overall sample structure is a superposition of deformed grains and DRXed grain managers. At a strain rate of 0.001 s^−1^, the alloy is fully recrystallized, and its texture is randomized and mainly determined by the DRXed grains. Therefore, the observed texture is closely influenced by the deformed and DRXed grains.

**Figure 16:**
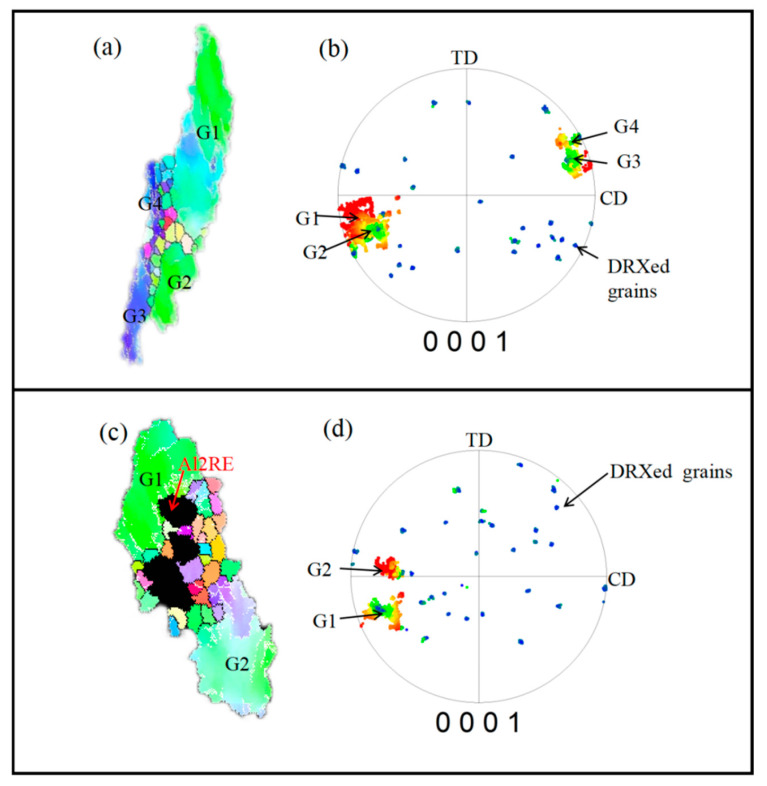
(**a**) IPF map and (**b**) {0001} pole figure of the typical microstructure of dynamic recrystallization; (**c**) IPF map and (**d**) {0001} pole figure of the typical microstructure of particle-stimulated nucleation.

The effects of DDRX and PSN on texture were clarified by studying the orientation relationship between the deformed and DRXed grains. Figure 16a,b presents the typical microstructure of DRXed grains formed through DDRX at the original grain boundaries and triple junctions, accompanied by grain boundary bowing. The orientation of the DRXed grains is highly random and notably differs from that of the deformed grains. In the microstructure of DRXed grains formed through PSN (Figure 16c,d), the DRXed grains are clustered around the secondary phase and exhibit no distinct orientation. As the orientations of the newly nucleated DRXed grains and deformed grains are independent, the recrystallization process provides a completely different set of orientation nuclei, leading to gradual diffusion of the orientations and a decreasing texture intensity with the proceeding of recrystallization.

## 4. Conclusions

In this work, isothermal compression experiments were performed on a Mg-15Gd-8Y-6Al-0.3Mn alloy with an elastic modulus of 51.3 GPa, which contains a substantial volume of Al_2_RE phases. The characteristics of flow stress and microstructural evolutions were analyzed, and thermal processing diagrams were established. The main conclusions are summarized below.

1. The flow curve of the alloy initially increased and then declined as the softening influence of DRX and DRV counterbalanced the strain-hardening phenomenon. Meanwhile, increasing the temperature while reducing the strain rate progressively reduced the peak stress.
2. From the hot compression tests, the constitutive model of hot deformation of the material was determined as follows:
  $$\dot{\varepsilon }=3.0052\times 10^{13}{\left[\sinh(0.0139\sigma )\right]}^{3.5840}\times \exp(-\frac{206.1}{RT})$$
3. From the thermal processing diagram, it was inferred that the instability zone increases with increasing strain and that instability zones predominate in the low-temperature, high-rate region. The optimum thermal processing region of the alloy is 350–500 °C in the 0.001–0.016 s^−1^ range of strain rates.
4. The DRX ratio and, correspondingly, the grain size increases with increasing temperature or decreasing strain rate. In addition, with the proceeding of recrystallization, the texture reduces the texture strength. Concurrently, particle-stimulated nucleation and discontinuous dynamic recrystallization are both verified to be responsible for the recrystallized microstructure of the alloy.

## Figures and Tables

**Table 1:** Chemical constituents of the alloy.

Nominal Constitution of the Alloy (wt.%)	Actual Chemical Composition of the Alloy (wt.%)				
	Gd	Y	Al	Mn	Mg
Mg-15Gd-8Y-6Al-0.3Mn	14.8	7.8	5.97	0.29	Bal.

**Table 2:** Parameters of the Mg alloys.

*n*_1_	*β*	*α* (MPa^−1^)	*n*	*Q* (KJ.mol^−1^)	*A*
5.2451	0.0731	0.0139	3.5840	206.1	3.0052 × 10^13^

## Data Availability

The original contributions presented in the study are included in the article, further inquiries can be directed to the corresponding author.

## References

[1] Wei S.H., Wang X.J., Li X.J., Shi H.L., Hu X.S., Xu C. (2024). Controlled preparation of a novel GNP@ MgO particles and its refinement mechanism in Mg-9Al alloy. J. Magnes. Alloys.

[2] Choi S.H., Kim D.H., Park S.S., You B.S. (2010). Simulation of stress concentration in Mg alloys using the crystal plasticity finite element method. Acta Mater..

[3] Zhou G., Yang Y., Luo Y.Y., Li Q., Luo Q., Zhang Y., Jiang B., Peng X.D., Pan S. (2023). Synergistic improvement of strength and plasticity of Mg-6Li-3Al-1Sn alloy by microstructure regulation via rotary swaging. Mater. Res. Lett..

[4] Xu C., Nakata T., Fan G.-H., Yamanaka K., Tang G.-Z., Geng L., Kamado S. (2019). Effect of Partially Substituting Ca with Mischmetal on the Microstructure and Mechanical Properties of Extruded Mg-Al-Ca-Mn based Alloys. Acta Metall. Sin. Engl. Lett..

[5] Liu X., Zhu B.W., Huang G.J., Li L.X., Xie C., Tang C.P. (2016). Initiation and strain compatibility of connected extension twins in AZ31 magnesium alloy at high temperature. Mater. Charact..

[6] Zhao L., Zha M., Gao Y., Guan K., Chen P., Zhang M.-N., Hua Z.-M., Jia H.-L., Wang H.-Y. (2023). Twinning-mediated plasticity by a novel multistage twinning mode in an Mg-Al-Gd alloy. Mater. Res. Lett..

[7] Xu C., Nakata T., Qiao X.G., Zheng M.Y., Wu K., Kamado S. (2017). Effect of LPSO and SFs on Microstructure Evolution and Mechanical Properties of Mg-Gd-Y-Zn-Zr. Sci. Rep..

[8] Zha M., Wang S.Q., Wang T., Jia H.L., Li Y.K., Hua Z.M., Guan K., Wang C., Wang H.Y. (2023). Developing high-strength and ductile Mg-Gd-Y-Zn-Zr alloy sheet via bimodal grain structure coupling with heterogeneously-distributed precipitates. Mater. Res. Lett..

[9] Wang X.J., Hu X.S., Wu K., Zheng M.Y., Zheng L., Zhai Q.J. (2009). The interfacial characteristic of SiCp/AZ91 magnesium matrix composites fabricated by stir casting. J. Mater. Sci..

[10] Shi H.L., Chao X., Hu X.S., Gan W.M., Wu K., Wang X.J. (2022). Improving the Young’s modulus of Mg via alloying and compositing—A short review. J. Magnes. Alloy..

[11] Zhao D., Chen X.H., Yuan Y., Pan F.S. (2020). Development of a novel Mg-Y-Zn-Al-Li alloy with high elastic modulus and damping capacity. Mater. Sci. Eng. A.

[12] Zhao D., Chen X., Ye J., Chen T., Dai Y., Liu C., Luo Z., Gao S., Zhang J., Yao J. (2019). Simultaneously improving elastic modulus and damping capacity of extruded Mg-Gd-Y-Zn-Mn alloy via alloying with Si. J. Alloys Compd..

[13] Ding C., Gam W.M., Hu X.S., Wu K., Wang X.J. (2020). Investigation into the influence of carbon nanotubes addition on residual stresses and mechanical properties in the CNTs@ SiCp/Mg-6Zn hybrid composite using neutron diffraction method. Mater. Sci. Eng. A.

[14] Li X.J., Wang X.J., Hu X.S., Xu C., Shao W.Z., Wu K. (2023). Direct conversion of CO_2_ to graphene via vapor-liquid reaction for magnesium matrix composites with structural and functional properties. J. Magnes. Alloy..

[15] Dong X., Feng L., Wang S., Ji G., Addad A., Yang H., Nyberg E.A., Ji S. (2022). On the exceptional creep resistance in a die-cast Gd-containing Mg alloy with Al addition. Acta Mater..

[16] Qiu D., Zhang M.X. (2014). The nucleation crystallography and wettability of Mg grains on active Al_2_Y inoculants in an Mg-10 wt% Y Alloy. J. Alloys Compd..

[17] Wang D.X., Jing Y., Lin B.S., Li J.P., Shi Y., Misra R.D.K. (2022). On the structure, mechanical behavior, and deformation mechanism of AZ91 magnesium alloy processed by symmetric and asymmetric rolling. Mater. Charact..

[18] Wang X.J., Liu W.Q., Hu X.S., Wu K. (2018). Microstructural modification and strength enhancement by SiC nanoparticles in AZ31 magnesium alloy during hot rolling. Mater. Sci. Eng. A.

[19] Jiang M.G., Xu C., Nakata T., Yan H., Chen R.S., Kamado S. (2016). Rare earth texture and improved ductility in a Mg-Zn-Gd alloy after high-speed extrusion. Mater. Sci. Eng. A.

[20] Kim W.J., Chung S.W., Chung C.S., Kum D. (2001). Superplasticity in thin magnesium alloy sheets and deformation mechanism maps for magnesium alloys at elevated temperatures. Acta Mater..

[21] Xi B.L., Fang G., Xu S.W. (2018). Multiscale mechanical behavior and microstructure evolution of extruded magnesium alloy sheets: Experimental and crystal plasticity analysis. Mater. Charact..

[22] Sahoo B.N., Panigrahi S.K. (2016). Synthesis, characterization and mechanical properties of in-situ (TiC-TiB2) reinforced magnesium matrix composite. Mater. Des..

[23] Tan C.T., Zou J., Wang D., Ma W.Y., Zhou K.S. (2022). Duplex strengthening via SiC addition and in-situ precipitation in additively manufactured composite materials. Compos. Part B Eng..

[24] Hu X., Sun Z., Zhang C., Wang X., Wu K. (2018). Microstructure and mechanical properties of bio-inspired Cf/Ti/Mg laminated composites. J. Magnes. Alloy..

[25] Romanova V.A., Balokhonov R.R., Schmauder S. (2009). The influence of the reinforcing particle shape and interface strength on the fracture behavior of a metal matrix composite. Acta Mater..

[26] Wang T., Chen Y.Z., Ouyang B., Zhou X., Hu J., Le Q.C. (2021). Artificial neural network modified constitutive descriptions for hot deformation and kinetic models for dynamic recrystallization of novel AZE311 and AZX311 alloys. Mater. Sci. Eng. A.

[27] Xu Y., Chen C., Zhang X.X., Dai H.H., Jia J.B., Bai Z.H. (2018). Dynamic recrystallization kinetics and microstructure evolution of an AZ91D magnesium alloy during hot compression. Mater. Charact..

[28] Zhu L.M., Li Q.N., Chen X.Y., Zhang Q. (2021). Effect of Sm on dynamic recrystallization of Mg-8Gd-0.5 Zr alloy during hot compression. J. Alloys Compd..

[29] Liu X.Q., Liu F., Liu Z.L., Xie H.J., Li J. (2020). Crystal structure, phase content, and tensile properties of As-cast Mg-Gd-Y-Al alloys. Mater. Today Commun..

[30] Fu Y.K., Wang L.P., Feng Y.C., Wang L., Zhao S.C. (2023). Effect of cooling rate on microstructure and mechanical properties of Mg-9Gd-4Y-1Zn-1Al alloy. Mater. Sci. Technol..

[31] Yuan S., Wang J.H., Zhang L., Jin P.P. (2023). Revealing the deformation behavior and microstructure evolution in Mg-12Y-1Al alloy during hot compression. J. Alloys Compd..

[32] Liu X., Yang H., Zhu B.W., Wu Y., Liu W., Tang C. (2022). Unveiling the mechanical response and accommodation mechanism of pre-rolled AZ31 magnesium alloy under high-speed impact loading. J. Magnes. Alloys.

[33] Niu Y.X., Hou J., Ning F.K., Chen X.R., Jia Y.H., Le Q.C. (2020). Hot deformation behavior and processing map of Mg-2Zn-1Al-0.2 RE alloy. J. Rare Earths.

[34] Zhang Q., Li Q.N., Chen X.Y., Bao J., Chen Z.Y. (2021). Effect of Sn addition on the deformation behavior and microstructural evolution of Mg-Gd-Y-Zr alloy during hot compression. Mater. Sci. Eng. A.

[35] Choi S.H., Kim J.K., Kim B.J., Park Y.B. (2008). The effect of grain size distribution on the shape of flow stress curves of Mg–3Al–1Zn under uniaxial compression. Mater. Sci. Eng. A.

[36] CSellars M., McTegart W.J. (1966). On the mechanism of hot deformation. Acta Met..

[37] Li X., Le Q., Li D., Wang P., Jin P., Cheng C., Chen X., Ren L. (2021). Hot tensile deformation behavior of extruded LAZ532 alloy with heterostructure. Mater. Sci. Eng. A.

[38] Zhang L., Yuan S., Wang J.H., Chen L.J., Jin P.P. (2022). Hot deformation behavior, processing map, microstructure evolution and dynamic recrystallization mechanism of Mg-5Al-0.6 Sc alloy. J. Alloys Compd..

[39] Zhang L., Zhang H.G., Liu Y.Z., Yuan S., Wang J.H., Chen L.J., Jin P.P. (2023). Revealing the dynamic recrystallization mechanism, extrusion deformation mechanism, and tensile deformation behavior of Mg-6Al-1Zn-1.1 Sc alloy. J. Mater. Res. Technol..

[40] Zener C., Hollomon J.H. (1944). Effect of strain rate upon plastic flow of steel. J. Appl. Phys..

[41] Prasad Y.V.R.K., Seshacharyulu T.J.M.R. (1998). Modelling of hot deformation for microstructural control. Int. Mater. Rev..

[42] Prasad Y.V.R.K., Gegel H.L., Doraivelu S.M., Malas J.C., Morgan J.T., Lark K.A., Barker D.R. (1984). Modeling of dynamic material behavior in hot deformation: Forging of Ti-6242. Metall. Trans. A.

[43] Prasad Y.V.R.K., Seshacharyulu T. (1998). Processing maps for hot working of titanium alloys. Mater. Sci. Eng. A.

[44] Srinivasan N., Prasad Y.V.R.K., Rao P.R. (2008). Hot deformation behaviour of Mg-3Al alloy—A study using processing map. Mater. Sci. Eng. A.

[45] Li Q.K., Yan H., Liu H.H., Chen R.S. (2022). Dynamic recrystallization mechanism and near-isotropic mechanical properties of WE43 magnesium alloy sheets rolled at different temperatures. Mater. Charact..

[46] Xia X.S., Chen Q., Zhao Z.D., Ma M.L., Li X.G., Zhang K. (2015). Microstructure, texture and mechanical properties of coarse-grained Mg-Gd-Y-Nd-Zr alloy processed by multidirectional forging. J. Alloys Compd..

[47] Xiao H.C., Tang B., Liu C.M., Gao Y.H., Yu S.L., Jiang S.N. (2015). Dynamic precipitation in a Mg-Gd-Y-Zr alloy during hot compression. Mater. Sci. Eng. A.

[48] Wang X.J., Wang X.M., Hu X.S., Wu K. (2020). Effects of hot extrusion on microstructure and mechanical properties of Mg matrix composite reinforced with deformable TC4 particles. J. Magnes. Alloys.

